# Outcome measurement for multiple long-term conditions research across health-care settings – an overview of reviews

**DOI:** 10.1186/s12916-026-04970-1

**Published:** 2026-06-19

**Authors:** Lauren L. O’Mahoney, Arron Peace, Clare L. Gillies, Ash Routen, Patrick J. Highton, Paul Bedford, Ruksar Abdala, Harini Sathanapally, Yogini V. Chudasama, Riya Patel, Rafael Perera, Sarah E. Hughes, Heather Walker, Mark P. Funnell, Kamlesh Khunti

**Affiliations:** 1 Leicester Diabetes Centre, University of Leicester, Leicester, UK; 2 NIHR Applied Research Collaboration East Midlands, Leicester, UK; 3 Leicester Real World Evidence Unit, University of Leicester, Leicester, UK; 4 Nuffield Department of Primary Care Health Sciences, University of Oxford, Oxford, UK; 5 Centre for Patient Reported Outcome Research, University of Birmingham, Birmingham, UK; 6 School of Cardiovascular & Metabolic Health, University of Glasgow, Glasgow, UK

**Keywords:** Multiple long-term conditions, core outcome sets, outcome measurement, overview of reviews, patient-reported outcomes

## Abstract

**Background:**

Multiple long-term conditions (MLTC) represent a growing global public health challenge, yet research is hindered by inconsistent outcomes across health-care settings and populations. Although several core outcome sets (COS) have been developed, important gaps remain in their coverage, inclusivity, and measurement validity. This overview of reviews summarised outcomes reported in systematic reviews of MLTC research to identify existing domains, describe how measurement instruments were addressed, and highlight areas requiring standardisation.

**Methods:**

We conducted a prospectively registered overview of reviews (PROSPERO CRD420251005152), following PRISMA, PRIOR, and SWiM guidelines. Eligible reviews included adults or children with MLTC—defined as the coexistence of two or more chronic conditions—and reported on outcome measures or COS relevant to this population. Five databases (MEDLINE, CINAHL, Scopus, Cochrane Library, COMET (Core Outcome Measures in Effectiveness Trials)) were searched from inception to April 2025. Two reviewers independently screened, extracted data, and appraised quality using the Joanna Briggs Institute checklist. A narrative synthesis mapped outcomes by domain (clinical, patient-reported, service/system, engagement/experience), care setting, and population subgroup.

**Results:**

From 6331 records, 10 reviews met inclusion criteria, encompassing 709 primary studies. Four developed COS, and six mapped outcomes without proposing COS. Quality of life and health-care utilisation were most consistently reported; treatment burden, patient engagement, and child outcomes were infrequently assessed. Existing COS advanced standardisation but remained limited in stakeholder diversity, geographic scope, and specification of measurement instruments, with only one including low- and middle-income countries.

**Conclusions:**

Greater inclusivity, validation, and global applicability are needed to operationalise agreed domains and improve comparability across MLTC research.

## Background

The co-existence of two or more long-term health conditions, termed multiple long-term conditions (MLTC), is a global public health challenge [1, 2]. In England, approximately 15% of the population currently lives with MLTC, and projections suggest that by 2035, nearly two-thirds of adults over the age of 65 will be affected [3, 4]. Living with MLTC is associated with reduced quality of life, increased mortality, and elevated healthcare utilisation across primary, secondary, and dental care settings [5, 6]. This places a major burden on patients and carers and strains healthcare systems, highlighting the need for coordinated research and policy responses [7]. Earlier challenges centred on which conditions to include, addressed by Delphi consensus [8], but uncertainty still remains regarding key outcome sets. Selecting appropriate outcomes is essential for validity, cross-study comparability, and utility of findings [9].

However, existing outcome frameworks are often fragmented and designed for specific disease combinations or healthcare contexts such as primary care, older adult populations, or single-country studies in high-income settings, and therefore limited in their applicability across diverse healthcare systems and populations, particularly in low- and middle-income countries [10]. Although a core outcome set (COS) for MLTC research was proposed in 2018, the development was restricted to high-income countries and primary care contexts, limiting its generalisability [11]. More recently, the COSMOS study has successfully developed COS for trials aimed at preventing and treating MLTC in low- and middle-income countries (LMIC) [12]. It incorporates outcomes such as quality of life, treatment adherence, adverse events, and health-related behaviours. While this represents an important advancement in extending COS development beyond high-income settings, gaps remain across the broader MLTC COS literature. Existing COS often focus on narrow clinical contexts or specific populations, such as older adults with polypharmacy [13], adults with type 2 diabetes and severe mental illness [14], or pregnant women with multiple conditions [15].

Few COS comprehensively capture the full spectrum of outcome domains, including patient-reported outcomes, health system performance, and treatment effectiveness across varied care settings, study designs, and socio-demographic groups. This lack of a universally applicable outcome set limits comparability across studies, impedes meaningful evidence synthesis, and hinders the development of effective interventions. It also limits the ability to compare findings across studies, as outcomes are often variably defined and grouped into overlapping categories, a form of outcome clustering that further complicates synthesis. Recognising these limitations, the National Institutes of Health has called for greater standardisation in MLTC outcome measurement [16]. Yet, despite this call to action, there remains a paucity of comprehensive evaluations that map the breadth of outcome measures currently in use and identify where COS are absent or underdeveloped.

An overview of reviews approach was chosen because multiple systematic reviews already exist in this field. Synthesising across them provides higher-level insights into outcome measurement in MLTC research across multiple studies and settings that cannot be obtained from individual reviews [17, 18]. This approach is particularly well-suited to the complex and heterogeneous patterns of MLTC, allowing for the evaluation of outcome measures spanning patient-reported outcomes, health system metrics, and treatment efficacy across varied care environments and demographic groups. Importantly, this review synthesises existing outcomes and also exposes gaps, such as underrepresented health domains, care settings, and population groups, where COS remain absent or insufficient. As the number of people living with MLTC continues to rise, addressing these omissions is essential to inform future research and support the development of more inclusive and context-sensitive outcome frameworks.

By synthesising existing systematic reviews, this overview aims to: (1) identify and map outcome domains reported in MLTC research; (2) examine how these outcomes are measured, including the use of core outcome sets; and (3) identify gaps in outcome coverage across populations, settings, and domains. This will contribute to the development of a more consistent and comprehensive approach to outcome measurement with implications for clinical practice, policy, and study design.

## Methods

### Study design and registration

This study was an overview of reviews, following the framework outlined by Belbasis et al. (2022) where applicable [17], which is designed to synthesise evidence from multiple systematic reviews and meta-analyses. This overview was conducted and reported in accordance with the Preferred Reporting Items for Overviews of Reviews (PRIOR) guidelines (Additional file 1: Table 1). PRISMA guidance was followed to support reporting of the study selection process, including the use of a flow diagram, and synthesis without meta-analysis (SWiM) guidance informed the narrative synthesis [19]. The review was prospectively registered with the International Prospective Register of Systematic Reviews (registration number: CRD420251005152).

### Search strategy and selection criteria

We included systematic reviews that examined outcome measures relevant to individuals living with MLTC, defined as the co-existence of two or more chronic conditions [2]. For the purpose of this overview, a systematic review was defined as a study that employed a structured and reproducible search strategy across one or more databases, with clearly defined inclusion and exclusion criteria, and a transparent process for study selection, data extraction, and synthesis.

#### Inclusion criteria

Eligible reviews could include participants of any age and were required to focus on populations consistent with the broad concept of MLTC. The definition proposed by Ho et al. (2022) [8], which provides a consensus list of 35 chronic conditions considered under the MLTC umbrella, was used as a guiding framework for data extraction and interpretation rather than as an exclusion criterion. The full list of conditions is provided in Additional file 1: Table 2.

Reviews were considered MLTC-specific if they explicitly examined populations with two or more long-term conditions or used terminology consistent with multimorbidity or multiple long-term conditions, rather than single-disease comorbidity. Reviews that focused on specific condition pairs were included only when the primary aim was to inform core outcome set development. In line with PRIOR guidance, eligibility was applied at the level of systematic reviews rather than primary studies.

#### Exclusion criteria

Narrative, scoping, and integrative reviews were excluded because they do not typically employ fully systematic and reproducible methods, such as comprehensive database searching, explicit eligibility criteria, duplicate screening, and structured data extraction, which are required to ensure methodological rigour and consistency in overviews of reviews. This is consistent with eligibility criteria applied in previous overviews of systematic reviews [20–22]. Reviews were included if they reported any core health outcomes, outcome sets, or recommended outcome domains relevant to MLTC research. We excluded any systematic reviews that did not provide information on core health outcomes or recommended domains for future research. Only English-language publications were considered eligible for inclusion. There were no limits on publication date or setting in which studies within the reviews were conducted, but only peer-reviewed systematic reviews published in English were included. Conference abstracts and articles for which the full text was not available were excluded.

A comprehensive search strategy was developed in consultation with an academic librarian. Multiple relevant electronic databases were searched from inception to 1st April 2025, including MEDLINE, Cumulative Index to Nursing and Allied Health Literature, Scopus, Cochrane Library, and the Core Outcome Measures in Effectiveness Trials Initiative. Search terms combined concepts for study design (e.g., *systematic review*, *meta-analysis*, *umbrella review*), MLTC (e.g., *multimorbid*, *multiple chronic conditions*, *syndemic*), and outcome measurement (e.g., *core outcome*, *International Consortium for Health Outcomes Measurement*, *COMET*, *Delphi technique*, *consensus*). The search strategies are provided in Additional file 1: Table 3–7.

### Data extraction and synthesis

All identified records were independently screened by two reviewers at both the title/abstract and full-text stages, with discrepancies resolved through discussion or, where necessary, consultation with a third reviewer. Reviewers were not blinded to article information (e.g., authors or journal) during screening. A list of studies excluded at the full-text stage, with reasons for exclusion, is provided in Additional file 1: Table 8. Data were extracted independently by two reviewers (LO and AP) using a pre-piloted, standardised Excel form. For each included review, we extracted information on the types of outcome domains reported (including patient-reported, health system, and treatment-related outcomes), whether and how stakeholders were involved in outcome selection, and the use of consensus-building methods such as Delphi or nominal group techniques. Additional contextual characteristics were also extracted, including participant age group (adult or child), socioeconomic and ethnic composition, care setting, and geographic region. Information related to the assessment of certainty or confidence in the evidence (e.g., use of consensus methods, COnsensus-based Standards for the selection of health Measurement INstruments (COSMIN) evaluations, or other appraisal approaches) was extracted where reported and considered narratively in the synthesis, as formal certainty grading frameworks were not applicable to this type of overview. Outcome domains were pre-specified and grouped into clinical, patient-reported, service/system, and engagement/experience categories to support consistent mapping across reviews. Where information was unclear or incompletely reported, this was interpreted based on the context provided within the review, and discrepancies were resolved through discussion between reviewers to ensure consistency. Information on whether and how included systematic reviews assessed the risk of bias or methodological quality of their primary studies (e.g., use of COSMIN or other appraisal tools) was also extracted and considered narratively in the synthesis.

Overlap of primary studies across included reviews was assessed by cross-referencing study identifiers (e.g., author names, publication year, and study characteristics) to identify duplicate inclusion. Where overlap was identified, this was documented and considered during synthesis to minimise double-counting of evidence. As the unit of analysis was outcome domains rather than specific intervention comparisons or effect estimates, overlap was not assessed at the level of individual comparisons or outcomes, and quantitative measures of overlap (e.g., corrected covered area) were not applied, as the degree of overlap was minimal and unlikely to influence the narrative synthesis If substantial overlap had been identified, we planned to prioritise the most comprehensive and methodologically robust reviews and to avoid double-counting by considering overlapping evidence only once within the synthesis.

Data were synthesised narratively following SWiM guidelines [19]. Where discrepancies in outcome reporting or classification were identified across reviews, these were resolved through discussion between reviewers and standardised through mapping to pre-specified outcome domains to ensure consistency in synthesis. Because our review focused on core outcome domains rather than quantitative effect estimates, statistical pooling through meta-analysis was neither feasible nor appropriate. Sensitivity analyses were not conducted, as the synthesis was descriptive and focused on mapping outcome domains rather than estimating effect sizes. This aligns with PRIOR guidance that quantitative synthesis in overviews is only appropriate when reviews are sufficiently homogenous; instead, a narrative approach was applied. Alternative synthesis methods (e.g., vote counting, harvest plots, or framework synthesis) were considered, but these approaches are designed for comparing intervention effects and were therefore not applicable to this review. A narrative approach was the most appropriate, as it allowed for mapping and summarising the outcomes reported across reviews. Findings were organised into pre-specified categories, including outcome domain (clinical, patient-reported, service/system, engagement/experience), care setting (primary, community, secondary/tertiary), study type (COS development vs non-COS), and socio-demographics (age, ethnicity, geographic/economic context). Heterogeneity across reviews (e.g., in populations, outcome definitions, and measurement approaches) was explored through stratification by outcome domain, care setting, and study type (COS vs non-COS).

A step-wise process was used: (1) charting extracted outcomes into the domain categories; (2) mapping outcomes against care setting, study type, and socio-demographic context; (3) constructing summary tables and evidence maps to visualise overlap and gaps; and (4) narratively synthesising patterns within and across categories. This structured approach enabled identification of areas of convergence, gaps, and underrepresented domains, highlighting priorities for the development of standardised, context-specific COS for MLTC research. The synthesis process was led by one reviewer (LO), with regular input from the wider review team, and key stages (including outcome domain mapping and categorisation) were independently checked by a second reviewer (AP), with discrepancies resolved through discussion to ensure consistency and validity.

### Quality assessment

The quality of included reviews was assessed using the Joanna Briggs Institute (JBI) Critical Appraisal Checklist for Systematic Reviews and Research Syntheses, which evaluates 11 key domains covering review design, methodological rigour, synthesis methods, and potential bias. The JBI Critical Appraisal Checklist was selected as it is suitable for appraising a broad range of systematic review types, including those without meta-analysis. Initial appraisal was conducted by one reviewer (LO) and independently checked by a second reviewer (AP), with discrepancies resolved through discussion. In accordance with the appraisal checklist, an overall numerical score was not generated, and no cut-off was applied. Instead, reviews were qualitatively categorised as higher quality when they met most of the JBI criteria (e.g., comprehensive search, transparent selection and extraction processes, appraisal of included studies) and as lower quality when these domains were absent or insufficiently reported. Assessment of review quality informed our narrative synthesis by highlighting domains where confidence in the evidence was greater (e.g., reviews with comprehensive search strategies and transparent methods) and where findings should be interpreted with caution. Risk of bias in primary studies was not reassessed; instead, we extracted and summarised the assessments reported within the included systematic reviews. This approach is consistent with guidance for overviews of reviews, which recommend relying on existing assessments to avoid duplication of effort and to reflect the level at which evidence is synthesised. Reporting bias due to missing results was not formally assessed separately; however, information on whether publication bias was assessed was extracted as part of the JBI appraisal and considered in the narrative synthesis.

### Patient and public involvement

People living with MLTC were engaged at three stages of this review. First, a focus group was held prior to study initiation to refine the research question and inform the development of the search strategy (n = 5). Following synthesis of the findings, a smaller follow-up consultation session (n = 2) was conducted to gather perspectives on the results, inform interpretation, and co-develop ideas for dissemination. Both participants had taken part in the initial focus group, and lower attendance reflected scheduling and availability constraints rather than disengagement. Due to the nature of the overview, patients were not recruited as study participants and were therefore not involved in assessing participant burden within the included reviews. Patient involvement is reported in line with the Guidance for Reporting Involvement of Patients and the Public 2 (GRIPP2) checklist [23]. The input from this group helped refine the research question by emphasising the importance of capturing patient-reported and experience-based outcomes, which informed the scope of outcomes included in this review.

## Results

### Study characteristics and grouping rationale

The flow of records through the screening and selection process is presented in Additional file 1: Fig. S1. The initial systematic database search yielded 6,331 records. Following title and abstract screening, 6,291 records were excluded. Forty full-text articles were assessed for eligibility, and thirty were excluded for reasons detailed in Additional file 1: Fig. S1, resulting in ten studies being included in the final synthesis. Across these ten systematic reviews, a total of 709 primary studies were identified. Overlap was minimal; only five studies [24–28] were included in more than one review, and all instances of overlap occurred exclusively between two reviews [29, 30].

As summarised in Table 1, four reviews developed COS [12–15], while six synthesised outcome measures without proposing a COS [29–34]. There was diversity in populations, from older adults with polypharmacy to pregnant women and adults with severe mental illness, across primary, community, hospital, and LMIC settings. The geographical scope of included studies also varied: some reviews incorporated evidence from multiple regions, including Europe, North America, and Asia [29–31], while others combined international systematic reviews with country-specific consensus processes [13, 14]. Of the ten reviews, nine were conducted wholly or predominantly in high-income country contexts. Only one review was specifically focused on LMICs [12], though some others included limited representation from middle-income settings[29, 31]. Consensus processes were most often centred in HIC, with the exception of one study which explicitly integrated stakeholders from LMICs [12].

**Table 1:** General characteristics of all included reviews (n = 10)

Author	Review type	Objective	Population	Setting	Geographical scope	No. of studies included	COS developed (Y/N)
Barello et al. [31]	Systematic review	To systematically review and appraise the psychometric properties of tools measuring patient engagement in adults with multimorbidity and their applicability for use within engagement programs	Adults with multimorbidity (≥3 long-term conditions)	Primary care and hospital settings	International (15 countries represented in the included studies – Brazil, Canada, China, Denmark, Germany, India, Iran, Italy, Netherlands, Norway, Saudi Arabia, Spain, Turkey, UK, United States)	23	N
Beuscart et al. [13]	COS Development Review	To develop a COS for clinical trials of medication review in multimorbid older patients with polypharmacy	Patients aged ≥65 years with polypharmacy (≥5 daily medications) and multimorbidity (≥2 chronic conditions)	Clinical trials in hospital, outpatient, and community settings	SR – Not reported Delphi – International (Australia, Belgium, Canada, France, Germany, Ireland, Italy, Netherlands, Norway, Switzerland, UK, USA). Face-to-face consensus meeting – Belgium	47	Y
Carswell et al. [14]	COS Development Review	To develop a core outcome set for use in effectiveness trials of diabetes self-management interventions in adults with both type 2 diabetes and SMI	Adults (18+) with type 2 diabetes and severe mental illness	Community and primary care	SR – International (Austria, Brazil, Canada, China, Finland, Germany, India, Iran, Israel, Japan, Korea, Mexico, Netherlands, Poland, Russia, South Africa, Sweden, Switzerland, Taiwan, US, Venezuela). Delphi and consensus - UK	54	Y
Huntley et al. [32]	Systematic review and guide	To identify measures of multimorbidity and morbidity burden suitable for use in research in primary care and community populations, and to investigate their validity in relation to anticipated associations with patient characteristics, process measures, and health outcomes.	“Predominantly based on adults”	Community and primary care	International (Australia, Canada, Europe, United States)	194 articles describing 184 studies	N
Lee et al. [33]	Systematic review	To list instruments for measuring levels of multimorbidity and their association with clinically important outcomes.	Community-dwelling adults	Primary care or general population	International (Asia, Europe, North America)	96	N
Lee et al. [15]	COS Development Review	To develop a core outcome set for studies of pregnant women with multimorbidity.	Pregnant women with multimorbidity	“It is not limited to specific long-term conditions, specific interventions or health care settings. The core outcome set would be applicable to observational and interventional studies.”	SR - Canada, Denmark, UK, USA. Consensus – International (Africa, Asia, Australia and New Zealand, Europe, Middle East, North America, South America)	26	Y
Møller et al. [34]	Systematic review	To search systematically for PROMS used among patients with multimorbidity. And evaluate the adequacy and validity of the PROMS identified.	Patients with multimorbidity	Primary Care	Not reported	101	N
Oemrawsingh et al. [29]	Systematic review	To identify general patient-reported morbidity instruments and evaluate their measurement properties for use in clinical practice and risk adjustment.	General adult populations with MLTC	Mixed (hospital, outpatient, community, primary care)	International (Australia, Belgium, Canada, Germany, India, Israel, Netherlands, Norway, Singapore, Spain, UK, United States)	34	N
Stirland et al. [30]	Systematic review	To identify and summarise multimorbidity indices that go beyond simple disease counts, assess inclusion of mental health, and provide guidance on index selection based on applicability, performance, and usage.	Adults (18+)	Community and population-level	International (Australia, Canada, Germany, India, Italy, New Zealand, Norway, Spain, Taiwan, UK, United States)	35	N
Vidyasagaran et al. [12]	COS Development Review	To develop two COS for future intervention studies relating to (1) prevention and (2) treatment of multimorbidity among adults residing in LMICs.	Adults (≥18 years) at risk of or living with multimorbidity	Community, primary care, and hospital settings in LMICs	SR - International (25 LMICs represented) Qualitative interviews - (Afghanistan, Bangladesh, Burkina Faso, Ghana, Mexico, Nepal, Nigeria, Pakistan, Peru, and Suriname Delphi and consensus - (lower LMICs, high income, upper middle income, and low-income countries.	109	Y (two COS: one for prevention, one for treatment)

*COS* core outcome set, *LMIC* low- and middle-income countries, *MLTC* multiple long-term conditions, *PROMs* patient reported outcome measures, *SMI* severe mental illness, *UK* United Kingdom.

### COS reviews

Table 2 summarises the four COS development studies. In addition, Fig. 2 illustrates the scope of these COS, mapping outcome domains against care settings. While the COS span primary, secondary, tertiary, community, and mixed settings, each was developed for a specific sub-population, meaning coverage remains fragmented rather than universal. All four combined systematic review evidence with consensus methods: three employed Delphi surveys, two included workshops or meetings, and one used both approaches sequentially. Each involved multiple stakeholder groups, typically patients, clinicians, and researchers, although the number and diversity of participants varied across studies. While all four COS studies defined outcome domains, only one also considered specific measurement instruments for selected outcomes such as medication appropriateness and quality of life [13]. The remaining COS did not undertake formal evaluation or recommendation of measurement tools, highlighting a continued gap between domain consensus and instrument standardisation.

**Table 2:** Outcomes of COS reviews (aligned with SWiM guidelines)

Author (Year)	Health Area / Scope	Stakeholder Involvement	PPIE Engagement	Consensus Method(s)	Outcome Domains	Time Points / Follow-up	Grouping Rationale	Synthesis Method	Standardised Metric Used	Strengths / Limitations
Beuscart et al. [13]	Medication review in multimorbid older adults with polypharmacy (≥65 years, ≥5 meds, ≥2 LTCs)	Patients, caregivers, healthcare professionals, researchers, policymakers (international)	Yes – patients and caregivers in interviews & consensus meetings	3-round Delphi+consensus meetings	3 domains: AE (e.g., drug-related hospitalisations, drug–drug interactions), Medication use (overuse, underuse, inappropriate meds), PROs (QoL, pain relief)	Not specified	Evaluative grouping across AE, medication use, PROs	Systematic review+qualitative interviews+Delphi+consensus	None; consensus-based COS	Strengths: international, rigorous multi-method approach, patient involvement; Limitations: protocol deviations, qualitative data from Belgium only, feasibility not assessed
Carswell et al. [14]	Self-management interventions for adults with severe mental illness (SMI)+type 2 diabetes	Patients, carers, health/social care staff, commissioners, academics; DIAMONDS Voice Panel	Yes – patients and carers throughout all stages (DIAMONDS Voice Panel)	Delphi+consensus workshop (≥70% agreement)	7 domains: HbA1c, blood pressure, body composition, QoL, diabetes self-management, diabetes distress, medication adherence	Not fully specified (trial context)	Evaluative – balance of biomedical and PROs	Literature review+stakeholder workshops+Delphi+consensus	None; consensus-based COS	Strengths: strong stakeholder engagement, inclusive Delphi process, filled evidence gap; Limitations: UK-only, limited generalisability, potential Delphi bias
Lee et al. [15]	Studies of pregnant women with multimorbidity (maternal & child outcomes)	Women, partners, health professionals, researchers	Yes – PPIE Advisory Group involved	Delphi (2 rounds)+consensus meetings	11 domains: Maternal (death, severe morbidity, change in LTCs, QoC/experience, new mental health conditions); Child (survival, neurodevelopment, gestational age, QoL, birth weight, separation from mother)	Not reported	Evaluative – maternal vs. child domains	Systematic review+focus groups+Delphi+consensus	COSMIN framework informed	Strengths: robust multi-stage process, international input, broad scope; Limitations: Delphi attrition, limited partner/child input, UK-centric consensus
Vidyasagaran et al. [12]	Interventions to prevent or treat multimorbidity in LMICs (COSMOS study)	People with multimorbidity, caregivers, researchers, clinicians, policymakers (LMICs & UK)	Yes – 4 PPIE members on steering committee & advisory panels	Modified Delphi+nominal group technique	Two COS: Prevention (AE, new comorbidity, health risk behaviours, QoL); Treatment (adherence, AE, out-of-pocket expenditure, QoL)	Not specified	Evaluative – prevention vs. treatment outcomes	Systematic review+qualitative interviews+Delphi+consensus	None; consensus-based COS	Strengths: COMET-guided, LMIC focus, mixed-methods, diverse stakeholder input; Limitations: Delphi only in English, underrepresentation of policymakers, generalisability beyond LMICs uncertain

*AE* adverse events, *COS* core outcome set, *COSMIN* consensus-based standards for the selection of health measurement instruments, *DIAMONDS* diabetes and mental illness, improving outcomes and self-management, *HbA1c* glycated haemoglobin, *LMIC* low and middle income countries, *LTC* long-term condition, *PPIE* patient and public involvement, *PPIE* patient and public involvement and engagement, *PROs* patient-reported outcomes, *QoC* quality of care, *QoL* quality of life, *SMI* severe mental illness, *UK* United Kingdom.

Beuscart and colleagues developed a COS for medication review in older adults with MLTC and polypharmacy [13]. The systematic review’s geographical scope was not reported, but the Delphi included experts from Europe, North America, and Australasia, and the final consensus meeting was held in Belgium. Seven outcomes were agreed across adverse events, medication use, and patient-reported domains. A second COS focused on pregnant women with MLTC [15]. Drawing on review evidence from Canada, Denmark, the UK, and the USA, this study combined systematic review findings with a global consensus process. Outcomes covered maternal mortality and morbidity, changes in long-term conditions, quality and experience of care, and child outcomes such as survival, neurodevelopment, and quality of life.

Another COS was designed for self-management interventions in adults with type 2 diabetes and severe mental illness [14]. The systematic review included studies from 20 countries across Europe, Asia, the Americas, and Africa, while consensus activities (Delphi and workshops) were UK-based. Seven outcomes were prioritised, including biomedical (e.g., HbA1c, blood pressure) and patient-reported measures (e.g., quality of life, diabetes distress, self-management). The fourth COS addressed MLTC prevention and treatment in LMICs [12]. This study combined a systematic review with qualitative interviews in 10 LMIC countries, followed by an international consensus phase with stakeholders from low-, middle-, and high-income settings. Outcomes for prevention included adverse events, incident comorbidity, risk behaviours, and quality of life, while treatment outcomes focused on adherence, adverse events, out-of-pocket costs, and quality of life. Together, COS reviews addressed clinical, service, and patient-centred domains, but varied in scope and representativeness. While some were global, others were restricted to the UK or Europe.

### Non-COS outcome reviews

Table 3 summarises six reviews that mapped or appraised existing instruments without proposing a COS. One review included engagement tools validated across 15 countries spanning Europe, Asia, and the Americas [31]. Half adopted broadly international perspectives [29, 30, 32] while Lee et al. [33] captured studies primarily from Asia, Europe, and North America [33]. By contrast, one study did not report the geographical scope of included studies [34]. Collectively, these reviews identified a wide range of measurement instruments, including multimorbidity indices (e.g., Charlson Comorbidity Index, Cumulative Illness Rating Scale, Adjusted Clinical Groups), quality-of-life measures (e.g., EQ-5D, SF-12, SF-36, WHOQOL-BREF), and patient-reported engagement and morbidity tools (e.g., Patient Activation Measure, Patient Health Engagement Scale, MULTIPleS). The EQ-5D and Charlson Comorbidity Index appeared most frequently across reviews as generic measures of health status and disease burden, respectively, although their content validity for MLTC populations was questioned. All six non-COS reviews undertook some degree of measurement instrument evaluation, assessing reliability, validity, or feasibility across clinical and patient-reported domains. However, each review identified considerable inconsistency in instrument use and limited cross-context applicability, underscoring the need for validated, multimorbidity-specific tools to operationalise agreed outcome domains. Further assessment of the psychometric performance or suitability of individual measurement tools was beyond the scope of this overview.

**Table 3:** Outcomes of non-COS outcome reviews (aligned with SWiM guidelines)

Author (Year)	Key Outcomes Reported	Time Points / Follow-up	Grouping Rationale	Synthesis Method	Standardised Metric Used	Certainty of Evidence	Strengths / Limitations	Presentation Format	Additional Information
Huntley et al. [32]	17 multimorbidity measures (e.g., Disease counts, Charlson Index, ACG, CDS, CIRS, DUSOI). Outcomes linked to patient characteristics, process measures, health outcomes.	Not applicable (measurement tool validity studies).	Grouped by measure type (simple counts vs. weighted indices vs. drug-based).	Descriptive comparative analysis.	Not standardised across studies.	Not assessed.	Strengths: Comprehensive mapping of tools; Limitations: High heterogeneity, no formal quality appraisal.	Narrative+tabular summary.	Found simple disease counts perform nearly as well as complex indices.
Stirland et al. [30]	35 multimorbidity indices beyond disease counts; outcomes: mortality (18), hospital admissions (13), healthcare use/costs (13), QoL (7), disability (7), mental health (9), self-rated health (5), drug use (4).	Varied (cross-sectional and longitudinal; e.g., 1–4 years).	Grouped indices by type (weighted counts, diagnostic categories, drug-based, physiological).	Narrative synthesis+quality assessment.	Not applicable.	Custom tool: 6 high, 22 satisfactory, 7 low quality.	Strengths: Inclusion of mental health, updated review; Limitations: Excluded grey literature, limited to full-text.	Narrative+evidence tables.	12 indices recommended for use. Lay contributor engaged in draft review.
Barello et al. [31]	8 patient engagement tools (PAM, PHE-S, PACIC, PPQ, PPET, PRE-HIT, HES, Small’s Scale); domains: engagement, empowerment, activation, participation.	Cross-sectional and longitudinal validation studies.	Grouped by instrument family and engagement construct.	Narrative synthesis+COSMIN appraisal.	COSMIN checklist.	Not explicitly reported.	Strengths: Conceptual synthesis, COSMIN evaluation; Limitations: Language restrictions, some tools under validation.	Narrative+psychometric evidence tables.	PAM and PHE-S strongest psychometric properties; no single tool covers all domains.
Oemrawsingh et al. [29]	10 patient-reported morbidity instruments (e.g., SR-CCI, SCQ, DBMA, CmSS). Outcomes: mortality, PROMS, healthcare utilisation.	Varied validation studies.	Grouped by instrument type and validation properties.	Narrative synthesis+psychometric evaluation.	Not applicable.	Not formally assessed.	Strengths: Identified widely used instruments; Limitations: Heterogeneity, search cut off 2018, no risk of bias assessment.	Tables+narrative summary.	SR-CCI and SCQ most cited; moderate predictive validity for mortality, weaker for utilisation.
Møller et al. [34]	6 PROMS for multimorbidity in primary care (HCHS, HCTD, PETS, MULTIPleS, TBQ, MTBQ). Focus: quality of life/illness perception/burden of treatment.	Not specified.	Grouped by PROM type.	COSMIN-based evaluation.	COSMIN checklist.	COSMIN appraisal: all PROMs had inadequacies.	Strengths: Used COSMIN, identified PETS as most adequate; Limitations: Limited PROMS, excluded psychiatric multimorbidity, search cut off 2018	Narrative+COSMIN appraisal tables.	Found no PROMS specifically measuring QoL in multimorbidity.
Lee et al. [33]	33 multimorbidity instruments; outcomes: mortality, QoL, healthcare use. Disease Count most common.	Varied follow-up.	Grouped using Sarfati framework (counts, indices, prescription-based, combinations).	Narrative synthesis+methodological appraisal.	Modified Newcastle-Ottawa Scale.	Quality varied across studies (details not summarised).	Strengths: Comprehensive and updated list; Limitations: English-only, excluded grey literature.	Narrative+tables.	Provides updated overview of tools; did not assess validity of instruments.

*ACG* adjusted clinical groups, *CDS* chronic disease score, *CIRS* cumulative illness rating scale, *CmSS* comorbidity symptom scale, *COSMIN* consensus-based standards for the selection of health measurement instruments, *DBMA* disease burden morbidity assessment, *HCHS* health care hassles scale, *HCTD* health care task difficulty, *HES* health engagement scale, *MTBQ* multimorbidity treatment burden questionnaire, *MULTIPleS* multimorbidity illness perceptions scale, *PACIC* patient assessment of chronic illness care, *PAM* patient activation measure, *PETS* patient experience with treatment and self-management, *PHE-S* patient health engagement scale, *PPET* patient preferences for engagement tool, *PPQ* patient participation questionnaire, *PRE-HIT* patient readiness to engage in health information technology, *PROMS* patient reported outcome measures, *QoL* quality of life, *SCQ* self-administered comorbidity questionnaire, *SR-CCI* self-reported Charlson comorbidity index, *TBQ* treatment burden questionnaire.

Huntley et al. [32] mapped 17 MLTC measures for use in primary and community care [32]. The review concluded that simple disease counts often performed comparably to more complex indices such as the Charlson Comorbidity Index and Adjusted Clinical Groups, although reliability and indexing limitations were noted. Lee and colleagues identified 33 instruments for measuring MLTC, with disease counts emerging as the most frequently applied approach [33]. These measures were most often examined in relation to mortality, quality of life, and healthcare utilisation. Stirland et al. [30] reviewed 35 MLTC indices and recommended 12 for application in research and practice [30]. Although most indices incorporated mental health, none adequately reflected the complex interactions between mental and physical conditions.

The remaining three reviews concentrated on patient-reported instruments. Oemrawsingh and colleagues evaluated 10 morbidity tools, which demonstrated moderate predictive validity for mortality but weaker associations with healthcare utilisation [29]. Møller et al. [34] assessed six PROMs (patient reported outcome measures) related to quality of life and burden of treatment in primary care, all of which exhibited methodological limitations [34]. Barello et al. [31] synthesised evidence on eight engagement measures, concluding that the Patient Activation Measure and Patient Health Engagement Scale displayed the strongest psychometric properties, although no single instrument captured the full construct of patient engagement [31].

Appraisal methods evolved over time, progressing from descriptive comparisons and bespoke tools in earlier reviews to COSMIN based evaluations in more recent studies. Despite this methodological development, certainty of evidence was rarely formalised, and important gaps remained, particularly regarding responsiveness, cross-cultural validity, and linkage to service-level outcomes.

### Synthesis method and standardised metrics

**Fig. 1:**
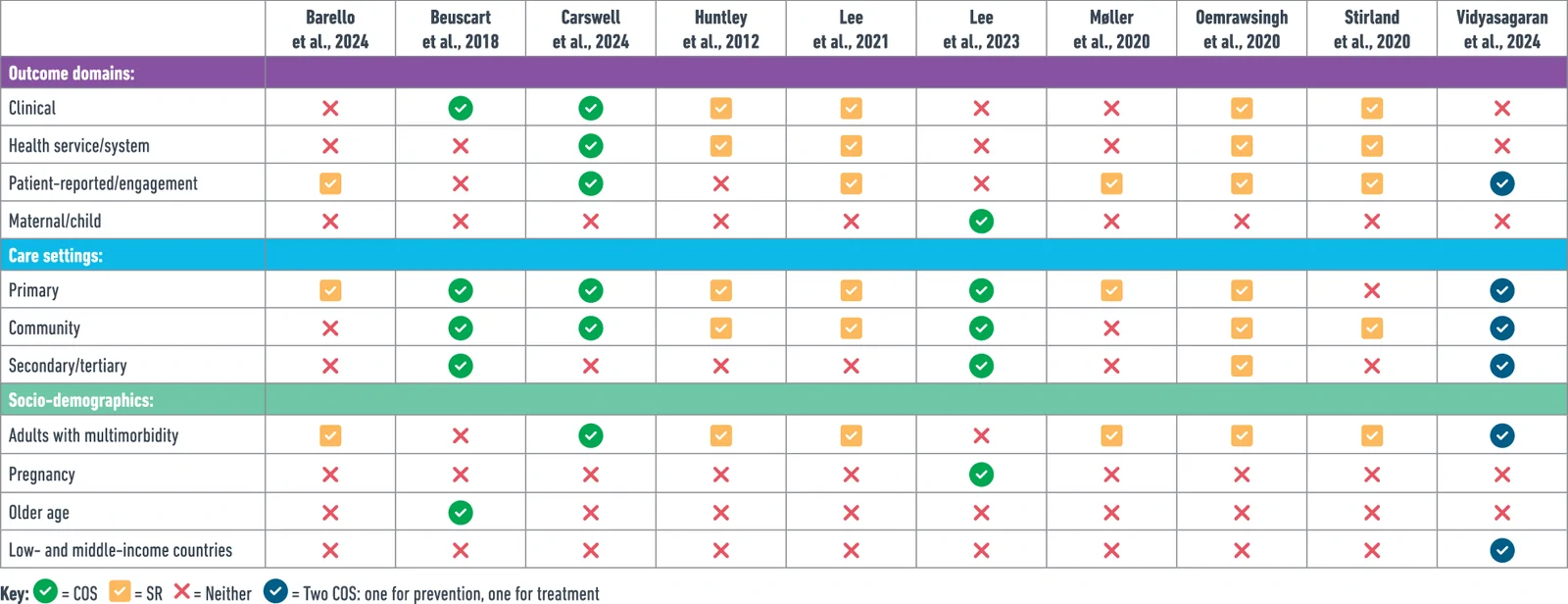
Matrix mapping outcome domains relevant to multiple long-term conditions (MLTCs) across healthcare settings and research methodologies. Each cell represents the presence or absence of outcome measures, supporting identification of evidence gaps and informing future research priorities. Key: Published COS (Tick and green) SR available (square and yellow) No COS or SR (cross and red)

Given marked heterogeneity in populations, instruments, and review aims, we used a narrative synthesis structured by the pre-specified grouping rationale. To enable comparability, we mapped the presence of outcome domains across reviews in summary tables and an evidence matrix to visualise overlap and gaps between COS and non-COS literature (Fig. 1; Additional file 1: Table 9). Meta-analysis and effect-direction plots were not applicable because the unit of analysis was the type of outcome/domain rather than intervention effects, and effect metrics were not comparable across instrument-focused reviews. Standardised metrics were generally not applicable for cross-review aggregation.

### Certainty of evidence and risk of bias

Certainty was rarely formalised. The COS development studies followed transparent consensus procedures with multi-stakeholder participation, which strengthen content validity of the resulting domains; however, none formally graded certainty of evidence for associations or responsiveness, and time points were often unspecified. Among non-COS reviews, instrument quality was appraised using COSMIN [31, 34] or bespoke/modified tools [30, 33], revealing variable methodological quality and frequent gaps in validation, especially for responsiveness, cross-cultural validity, and linkage to system-level outcomes. The overall confidence in the evidence across outcome domains is therefore moderate at best and constrained by heterogeneity in instruments and reporting. Using the JBI Critical Appraisal Checklist, all ten reviews met ten of the eleven quality criteria, indicating consistently high methodological standards. The only domain not routinely addressed was publication bias. Full domain-level assessments are provided in Additional file 1: Table 10. Potential reporting bias due to missing or incompletely reported results across included reviews was considered in the synthesis, particularly where publication bias was not assessed; this may have influenced the completeness of outcome reporting.

### Presentation of results

Findings are presented as structured summary tables for general characteristics and for COS versus non-COS outcomes, alongside a cross-review summary outcome domain table (Tables 1–3). To support interpretation, Fig. 1 presents a cross-review evidence map of all included studies (n = 10), showing the presence or absence of outcome measures across different domains, care settings, and study types (COS vs non-COS). This visual summary highlights areas of overlap and domains where evidence remains sparse. This approach enhances transparency of the grouping rationale, clarifies where domains converge across COS and non-COS literatures (e.g., quality of life, healthcare utilisation), and highlights underrepresented areas (e.g., child outcomes beyond pregnancy, treatment burden, engagement and experience measures outside mental health and diabetes contexts).

### Patient and public perspectives

Two patient and public contributors participated in a follow-up consultation session, providing insights into the relevance and interpretation of the review findings. Participants highlighted that quality of life was the most important and meaningful outcome, echoing its consistent reporting across both COS and non-COS reviews. They also emphasised that treatment burden and patient engagement outcomes were underrepresented, which directly reflect the gaps identified in our synthesis. A key concern raised was the need for outcome measures and future research to capture and reflect the experiences of people from all ethnicities, aligning with our finding that most existing reviews were based in high-income, predominantly White populations. Child and family outcomes were also considered important, reinforcing the limited attention to paediatric and intergenerational outcomes identified in included reviews. Overall, the group supported the need for more inclusive, patient-centred outcome measures and stressed the importance of co-developing future research priorities with people living with MLTC. These perspectives closely align with the findings of this review and reinforce the importance of prioritising patient-reported and experience-based outcomes in MLTC research. They also highlight the need for greater diversity and inclusivity in the development of core outcome sets, ensuring that outcome selection reflects the priorities and experiences of broader populations living with MLTC. Together, these insights underscore the value of meaningful patient and public involvement in shaping outcome measurement and research priorities.

### Integrated interpretation

Across all ten reviews, outcomes clustered into clinical, service/system, patient-reported, engagement/experience, mental health, and maternal/child domains. Quality of life and healthcare utilisation appeared most consistently across COS and non-COS reviews, while mortality and hospital admissions were common in measurement-focused syntheses. COS reviews made explicit progress in standardising what should be measured through consensus, notably by embedding patient-centred outcomes and, in the case of Lee et al. [15], extending to child outcomes. Non-COS reviews revealed persistent variability in how those outcomes should be measured, with no single instrument demonstrating comprehensive validity across populations and settings. Together, these findings underscore the need to pair consensus-defined domains with robust, responsive, and context-sensitive measurement strategies, particularly for patient engagement, treatment burden, and child outcomes, so that MLTC research can achieve both relevance and comparability across health care settings.

## Discussion

### Summary of key findings

This overview of reviews synthesised outcomes and outcome measurement instruments for MLTC research reported in 709 individual studies across ten systematic reviews, including four COS development studies. Outcomes were clustered into clinical, service/system, patient-reported, and engagement/experience. Quality of life and healthcare utilisation were consistently reported across both COS and non-COS reviews, whereas mortality, hospital admissions, and treatment burden were more context-dependent. COS development studies demonstrated progress in standardising outcomes through multi-stakeholder consensus, but non-COS reviews highlighted persistent heterogeneity in outcome selection, measurement tools, and longitudinal responsiveness, particularly in underrepresented populations such as children, socioeconomically disadvantaged groups, and people living in low- and middle-income countries, and across community and integrated care settings. This variation is not unexpected given the breadth of conditions encompassed within MLTC, inconsistencies in how MLTC is defined, and the continued tendency of healthcare systems to organise and evaluate care around single-disease models rather than multiple conditions.

### Comparison with previous literature

This review represents the first comprehensive synthesis of COS in MLTC research that spans diverse populations, healthcare settings, and socio-demographic groups. Previous COS efforts, such as COSmm (Core Outcome Set for Multimorbidity Research) [11], were largely confined to HICs and primary care contexts. More recent initiatives, including the COSMOS study, have expanded the scope to LMICs, incorporating outcomes relevant to both prevention and treatment, such as adverse events, health risk behaviours, and out-of-pocket expenditure [12]. Despite these advances, significant gaps remain. Notably, standardised measurement tools for patient engagement and treatment burden are underdeveloped. Barello et al. [31] found that while instruments like the Patient Activation Measure and Patient Health Engagement Scale show promise, none fully capture the complexity of engagement in MLTC research [31].

**Fig. 2:**
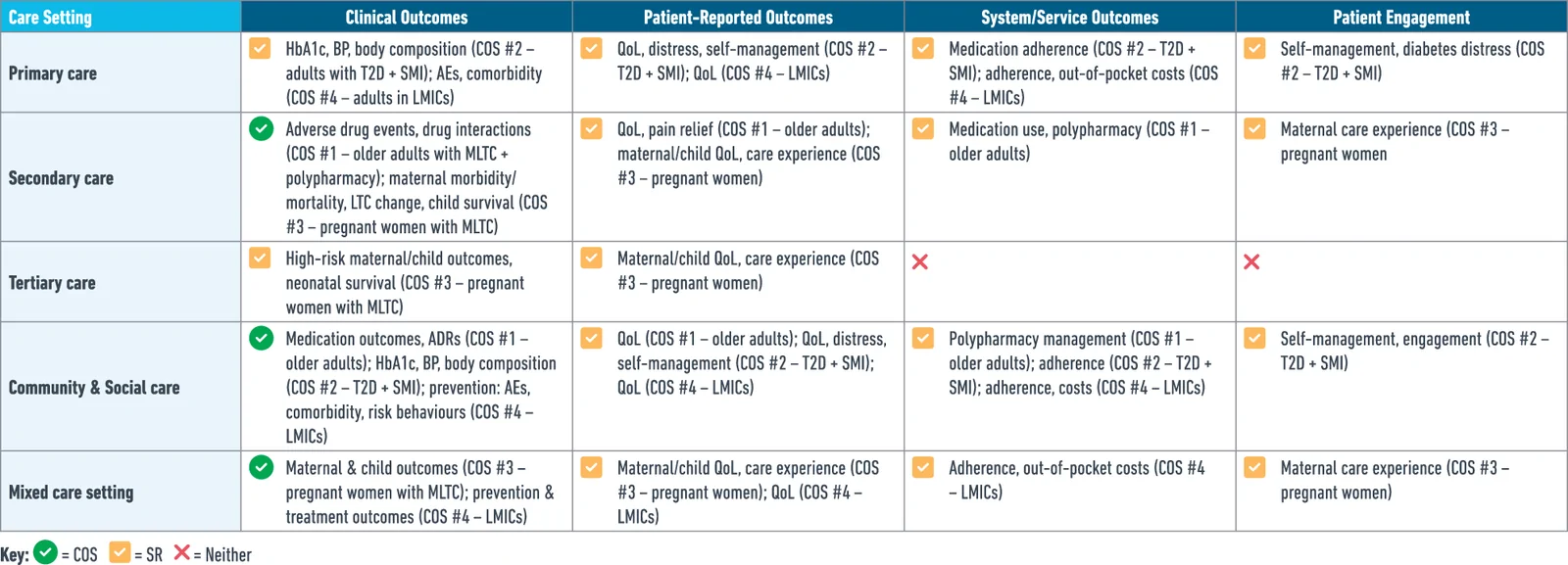
Matrix of outcome domains covered by existing core outcome sets (COS) for multiple long-term conditions (MLTC) across care settings. The matrix summarises the extent to which COS address four outcome domains (clinical, patient-reported, system/service, and patient engagement) across five types of care setting (primary, secondary, tertiary, community & social care, and mixed care). All COS were developed for specific sub-populations: older adults with MLTC and polypharmacy (COS-1), adults with type 2 diabetes and severe mental illness (COS-2), pregnant women with MLTC (COS-3), and adults in LMICs (COS-4). Thus, even where multiple COS overlap (orange), coverage remains restricted to these groups rather than universal to all people with MLTC

Child health outcomes beyond pregnancy also remain insufficiently represented. While Lee et al. [15] developed a COS for pregnant women with MLTC, including some child health metrics, broader paediatric outcomes in the context of familial or intergenerational MLTC are rarely considered [15]. Growing evidence underscores the importance of investigating early-life childhood MLTC, as adversities in this age group, including poor health, socioeconomic disadvantage, and psychosocial stress, can significantly amplify the risk of MLTC later in life and aggravate health inequalities over the life course [35, 36]. However, children and young people living with MLTC also have distinct and evolving care needs, often managing their own conditions and interacting with multiple services across health, education, and social care. This highlights the importance of developing outcome sets that are tailored to paediatric populations, capturing outcomes relevant to their current health, functioning, and lived experiences, rather than focusing solely on long-term trajectories into adulthood. Despite this, progress in this area is constrained by the absence of a clear consensus on how MLTC should be defined and measured in paediatric populations, and by the limited availability of validated outcome instruments, particularly patient-reported measures suitable for children and adolescents. These gaps echo calls from the National Institutes of Health (NIH) for greater harmonisation and inclusivity in outcome measurement. Recent NIH-funded initiatives aim to develop outcome-focused quality measures for mental health and MLTC, including tools for goal-directed care and personalised quality of life assessment [37]. Together, these findings underscore the need to pair consensus-defined domains with context-specific measurement strategies. Without further research on outcome development and consensus, MLTC research risks perpetuating fragmentation and limiting the comparability and applicability of findings across settings and populations. The evidence maps (Figs. 1 and 2) reinforce these findings, visually demonstrating outcome domain overlap (e.g., quality of life, healthcare utilisation) alongside major gaps (e.g., patient engagement, treatment/symptom burden, and child outcomes) across both COS and non-COS reviews.

### Strengths and limitations

This review benefits from a structured overview of reviews approach, adherence to PRIOR, PRISMA, and SWiM guidelines, and rigorous dual reviewer screening, extraction, and quality appraisal. By integrating COS and non-COS reviews, it provides a comprehensive map of outcomes across domains, populations, and settings. However, the review is limited by the small number of included reviews, English-language restriction, and heterogeneity in study design, instruments, and reporting. Limitations also arise from the synthesis method itself; narrative synthesis has the risk of selective emphasis. By reducing outcomes to presence/absence at the domain level, nuance in how instruments performed will not have been captured. Additional limitations specific to the overview of reviews approach include reliance on published systematic reviews (rather than primary studies), and potential omission of outcomes assessed in unpublished or ongoing reviews.

### Patient and public involvement: impact and reflections

Patient and public involvement had a tangible impact on this review. Early engagement helped shape the research question, while subsequent input informed interpretation of the findings and prioritisation of dissemination routes. These contributions ensured that the review remained aligned with the lived experiences and priorities of people with MLTC. A limitation of our PPIE approach was that involvement was limited to two focus groups drawn from a single regional population, which may restrict the diversity of perspectives. Future reviews should consider broader and more continuous engagement, including diverse socio-demographic groups and international perspectives, to strengthen the generalisability of patient-informed priorities.

### Implications for research, policy, and practice

The findings underscore the importance of pairing consensus-defined domains with context-sensitive measurement instruments to enable comparability and relevance across studies and settings. Although all included COS addressed outcomes relevant to people with multiple long-term conditions, one was condition-specific, focused on adults with type 2 diabetes and severe mental illness, rather than encompassing the broader multimorbidity spectrum. This distinction underlines the challenge of balancing condition-specific depth with cross-condition relevance in MLTC outcome research. Future MLTC research should prioritise: developing and validating measures for underrepresented domains such as treatment/symptom burden and patient engagement; expanding COS development to include children, diverse socio-economic and ethnic groups, and LMIC contexts; and incorporating longitudinal studies and feasibility assessment into COS.

As shown in Fig. 2, even where outcome domains appear to be included in more than one COS, applicability remains limited to specific populations, such as older adults with polypharmacy, pregnant women with MLTC, adults with type 2 diabetes and severe mental illness, or adults in LMICs. Development of a more generalisable COS for MLTC research across broader populations and settings is still needed. For clinical practice, these standardised outcomes will inform more consistent evaluation of interventions, quality improvement initiatives, and policy development across the care continuum.

## Conclusions

This overview of reviews demonstrates that while COS have advanced the standardisation of outcome selection in MLTC research, substantial gaps remain, particularly in underrepresented populations, care settings, and outcome domains. Addressing these gaps through context-sensitive, validated outcome measures is critical to improving comparability, relevance, and impact of future MLTC research.

## Supplementary information

Additional file 1: Supplementary materials supporting the findings of this overview of reviews. Fig. S1. Preferred Reporting Items for Systematic Reviews and Meta-Analyses (PRISMA) flow diagram of study selection. Table S1-S10.

## Data Availability

The datasets generated during the current review, including extracted data and data collection forms, are available from the corresponding author on reasonable request.

## Abbreviation

COS: Core outcome set
COSMIN: COnsensus-based Standards for the selection of health Measurement INstruments
JBI: Joanna Briggs Institute
LMIC: Low- and middle-income country
MLTC: Multiple long-term conditions
PPIE: Patient and public involvement and engagement
PRIOR: Preferred Reporting Items for Overviews of Reviews
PROMs: Patient-reported outcome measures
PRISMA: Preferred Reporting Items for Systematic Reviews and Meta-Analyses
PROSPERO: International Prospective Register of Systematic Reviews
SWiM: Synthesis Without Meta-analysis
