# Supplementary material for: Outcome measurement for multiple long-term conditions research across health-care settings – an overview of reviews
Source: BMC Med. 2026 Jun 19;24:473. doi: 10.1186/s12916-026-04970-1 (PMC13548526; doi:10.1186/s12916-026-04970-1)
Supplement: Supplementary file 1 [file 12916_2026_4970_MOESM1_ESM.docx]

**Identification of studies via databases and registers**

Records removed *before screening*:

Duplicate records removed
(n = 2,796)

Records marked as ineligible by automation tools (n = 0)

Records removed for other reasons (n = 0)

Records identified from*:

Databases (n = 9,127)

Medline (n = 2,742)

Scopus (n = 4,657)

Cochrane (n = 105)

CINAHL (n = 1,620)

COMET (n = 3)

**Identification**

Records screened

(n = 6,331)

Records excluded**

(n = 6,291)

Reports sought for retrieval

(n = 40)

Reports not retrieved

(n = 0)

**Screening**

Reports excluded: 30

No core outcome development (n = 19)

Wrong study design (n = 5)

Wrong outcomes (n = 2)

Wrong patient population (n = 2)

Duplicate (preprint) (n = 1)

Language (n = 1)

Reports assessed for eligibility

(n = 40)

Studies included in review

(n = 10)

**Included**

**Additional file 1: Fig. S1.** Preferred Reporting Items for Systematic Reviews and Meta-Analyses Flow Diagram.

**Additional file 1: Table 1.** PRIOR checklist for reporting of overviews of reviews.

| **Section**  **Topic** | **#** | **Item** | **Location**  **reported** |
| --- | --- | --- | --- |
| **TITLE** | | | |
| Title | 1 | Identify the report as an overview of reviews. | Page 1 |
| **ABSTRACT** | | | |
| Abstract | 2 | Provide a comprehensive and accurate summary of the purpose, methods, and results of the overview of reviews. | Pages 5-6 |
| **INTRODUCTION** | | | |
| Rationale | 3 | Describe the rationale for conducting the overview of reviews in the context of existing knowledge. | Pages 7-8 |
| Objectives | 4 | Provide an explicit statement of the objective(s) or question(s) addressed by the overview of  reviews. | Page 9 |
| **METHODS** | | | |
| Eligibility criteria | 5a | Specify the inclusion and exclusion criteria for the overview of reviews. If supplemental primary  studies were included, this should be stated, with a rationale. | Pages 9-10 |
|  | 5b | Specify the definition of ‘systematic review’ as used in the inclusion criteria for the overview of  reviews. | Pages 9-10 |
| Information sources | 6 | Specify all databases, registers, websites, organizations, reference lists, and other sources searched or consulted to identify systematic reviews and supplemental primary studies (if included). Specify the date when each source was last searched or consulted. | Page 11 |
| Search strategy | 7 | Present the full search strategies for all databases, registers and websites, such that they could be  reproduced. Describe any search filters and limits applied. | Supplementary Material |
| Selection process | 8a | Describe the methods used to decide whether a systematic review or supplemental primary study  (if included) met the inclusion criteria of the overview of reviews. | Pages 10-11 |
|  | 8b | Describe how overlap in the populations, interventions, comparators, and/or outcomes of  systematic reviews was identified and managed during study selection. | Pages 11-12 |
| Data collection process | 9a | Describe the methods used to collect data from reports. | Pages 11-12 |
|  | 9b | If applicable, describe the methods used to identify and manage primary study overlap at the level  of the comparison and outcome during data collection. For each outcome, specify the method used  to illustrate and/or quantify the degree of primary study overlap across systematic reviews. | Pages 11-12 |
|  | 9c | If applicable, specify the methods used to manage discrepant data across systematic reviews  during data collection. | Page 12 |
| Data Items | 10 | List and define all variables and outcomes for which data were sought. Describe any assumptions  made and/or measures taken to identify and clarify missing or unclear information. | Page 11-12 |
| Risk of bias assessment | 11a | Describe the methods used to assess risk of bias or methodological quality of the included  systematic reviews. | Page 14 |
|  | 11b | Describe the methods used to collect data on (from the systematic reviews) and/or assess the risk  of bias of the primary studies included in the systematic reviews. Provide a justification for  instances where flawed, incomplete, or missing assessments are identified but not re-assessed. | Page 14 |
|  | 11c | Describe the methods used to assess the risk of bias of supplemental primary studies (if included) | N/A |
| Synthesis methods | 12a | Describe the methods used to summarize or synthesize results and provide a rationale for the  choice(s). | Pages 12-13 |
|  | 12b | Describe any methods used to explore possible causes of heterogeneity among results. | Page 13 |
|  | 12c | Describe any sensitivity analyses conducted to assess the robustness of the synthesized results. | Page 12 |
| Reporting bias assessment | 13 | Describe the methods used to collect data on (from the systematic reviews) and/or assess the risk  of bias due to missing results in a summary or synthesis (arising from reporting biases at the levels  of the systematic reviews, primary studies, and supplemental primary studies, if included). | Pages 14-15 |
| Certainty assessment | 14 | Describe the methods used to collect data on (from the systematic reviews) and/or assess certainty  (or confidence) in the body of evidence for an outcome. | Pages 11-12 |
| **RESULTS** | | | |
| Systematic review and supplemental primary study selection | 15a | Describe the results of the search and selection process, including the number of records screened,  assessed for eligibility, and included in the overview of reviews, ideally with a flow diagram. | Supplementary Material (Fig 1) |
|  | 15b | Provide a list of studies that might appear to meet the inclusion criteria, but were excluded, with  the main reason for exclusion. | Page 11; Supplementary Material |
| Characteristics of systematic reviews and  Supplemental primary studies | 16 | Cite each included systematic review and supplemental primary study (if included) and present its  characteristics. | Pages 16-18; Tables 1-3 |
| Primary study overlap | 17 | Describe the extent of primary study overlap across the included systematic reviews. | Page 16; Supplementary Material |
| Risk of bias in systematic reviews, primary studies, and supplemental primary studies | 18a | Present assessments of risk of bias or methodological quality for each included systematic review | Pages 20-21; Supplementary Material |
|  | 18b | Present assessments (collected from systematic reviews or assessed anew) of the risk of bias of  the primary studies included in the systematic reviews. | Pages 20-21; Supplementary Material |
|  | 18c | Present assessments of the risk of bias of supplemental primary studies (if included). | N/A |
| Summary or synthesis of results | 19a | For all outcomes, summarize the evidence from the systematic reviews and supplemental primary  studies (if included). If meta-analyses were done, present for each the summary estimate and its  precision and measures of statistical heterogeneity. If comparing groups, describe the direction of  the effect. | Pages 16–22; Tables/Figures |
|  | 19b | If meta-analyses were done, present results of all investigations of possible causes of  heterogeneity. | N/A |
|  | 19c | If meta-analyses were done, present results of all sensitivity analyses conducted to assess the  robustness of synthesized results. | N/A |
| Reporting biases | 20 | Present assessments (collected from systematic reviews and/or assessed anew) of the risk of bias  due to missing primary studies, analyses, or results in a summary or synthesis (arising from  reporting biases at the levels of the systematic reviews, primary studies, and supplemental primary  studies, if included) for each summary or synthesis assessed. | Pages 20-21; Supplementary Material |
| Certainty of evidence | 21 | Present assessments (collected or assessed anew) of certainty (or confidence) in the body of  evidence for each outcome. | Pages 20-21 |
| **DISCUSSION** | | | |
| Discussion | 22a | Summarize the main findings, including any discrepancies in findings across the included  systematic reviews and supplemental primary studies (if included). | Pages 23-24 |
|  | 22b | Provide a general interpretation of the results in the context of other evidence. | Pages 23-24 |
|  | 22c | Discuss any limitations of the evidence from systematic reviews, their primary studies, and  supplemental primary studies (if included) included in the overview of reviews. Discuss any  limitations of the overview of reviews methods used. | Pages 25-26 |
|  | 22d | Discuss implications for practice, policy, and future research (both systematic reviews and  primary research). Consider the relevance of the findings to the end users of the overview of  reviews, e.g., healthcare providers, policymakers, patients, among others. | Pages 26-27 |
| **OTHER INFORMATION** | | | |
| Registration and protocol | 23a | Provide registration information for the overview of reviews, including register name and  registration number, or state that the overview of reviews was not registered. | Page 9 |
|  | 23b | Indicate where the overview of reviews protocol can be accessed, or state that a protocol was not  prepared. | Page 9 |
|  | 23c | Describe and explain any amendments to information provided at registration or in the protocol.  Indicate the stage of the overview of reviews at which amendments were made. | N/A |
| Support | 24 | Describe sources of financial or non-financial support for the overview of reviews, and the role of  the funders or sponsors in the overview of reviews. | Pages 28-29 |
| Competing interests | 25 | Declare any competing interests of the overview of reviews' authors. | Page 28 |
| Author information | 26a | Provide contact information for the corresponding author. | Page 1 |
|  | 26b | Describe the contributions of individual authors and identify the guarantor of the overview of  reviews. | Page 28 |
| Availability of data and other materials | 27 | Report which of the following are available, where they can be found, and under which conditions  they may be accessed: template data collection forms; data collected from included systematic  reviews and supplemental primary studies; analytic code; any other materials used in the overview  of reviews. | Page 28 |

(Gates M, Gates A, Pieper D, et al. Reporting guideline for overviews of reviews of healthcare interventions: development of the PRIOR statement. BMJ 2022;378:e070849. doi:10.1136/bmj-2022-070849.)

**Additional file 1: Table 2.** Conditions recommended for inclusion in multimorbidity measurement.

| **Body system** | **Conditions recommended for “always include” (n = 24)** | **Conditions recommended for “usually include” (n = 35) *** |
| --- | --- | --- |
| Cardiovascular disease | Stroke; coronary artery disease; heart failure; peripheral artery disease | Heart valve disorders; arrhythmia; venous thromboembolic disease; aneurysm; hypertension (treated and untreated) |
| Metabolic and endocrine disease | Diabetes; Addison’s disease; cystic fibrosis | Thyroid disorders |
| Respiratory disease | Chronic obstructive pulmonary disease; asthma | Bronchiectasis |
| Neurological disease | Parkinson’s disease; epilepsy; multiple sclerosis; paralysis | Transient ischaemic attack; peripheral neuropathy; chronic primary pain |
| Cancer | Solid organ cancers; haematological cancers; metastatic cancers | Melanoma; benign cerebral tumours causing disability |
| Mental and behavioural disorders | Dementia; schizophrenia | Depression; anxiety; bipolar disorder; drug or alcohol misuse; eating disorders; autism; post-traumatic stress disorder |
| Musculoskeletal disease | Connective tissue disease | Osteoarthritis; long-term musculoskeletal problems due to injury; osteoporosis; gout |
| Digestive disease | Chronic liver disease; inflammatory bowel disease | Chronic pancreatic disease; peptic ulcer |
| Urogenital disease | Chronic kidney disease; end-stage kidney disease | Endometriosis; chronic urinary tract infection |
| Haematological disease |  | Anaemia (including pernicious anaemia and sickle cell anaemia) |
| Eye disease |  | Vision impairment that cannot be corrected |
| Ear disease |  | Hearing impairment that cannot be corrected; Meniere’s disease |
| Infectious disease | HIV/AIDS | Chronic Lyme disease; tuberculosis; post-acute COVID-19 |
| Congenital conditions |  | Congenital disease and chromosomal abnormalities |

**Additional file 1: Table 3.** Medline Search Strategy**.**

| 1. | (systematic review or meta-analysis).pt. |
| --- | --- |
| 2. | meta-analysis/ or systematic review/ or systematic reviews as topic/ or meta-analysis as topic/ or "meta analysis (topic)"/ or "systematic review (topic)"/ or exp technology assessment, biomedical/ or network meta-analysis/ |
| 3. | ((systematic* adj3 (review* or overview*)) or (methodologic* adj3 (review* or overview*))).ti,ab,kf. |
| 4. | ((quantitative adj3 (review* or overview* or synthes*)) or (research adj3 (integrati* or overview*))).ti,ab,kf. |
| 5. | ((integrative adj3 (review* or overview*)) or (collaborative adj3 (review* or overview*)) or (pool* adj3 analy*)).ti,ab,kf. |
| 6. | (data synthes* or data extraction* or data abstraction*).ti,ab,kf. |
| 7. | (handsearch* or hand search*).ti,ab,kf. |
| 8. | (mantel haenszel or peto or der simonian or dersimonian or fixed effect* or latin square*).ti,ab,kf. |
| 9. | (met analy* or metanaly* or technology assessment* or HTA or HTAs or technology overview* or technology appraisal*).ti,ab,kf. |
| 10. | (meta regression* or metaregression*).ti,ab,kf. |
| 11. | (meta-analy* or metaanaly* or systematic review* or biomedical technology assessment* or bio-medical technology assessment*).mp,hw. |
| 12. | (medline or cochrane or pubmed or medlars or embase or cinahl).ti,ab,hw. |
| 13. | (cochrane or (health adj2 technology assessment) or evidence report).jw. |
| 14. | (comparative adj3 (efficacy or effectiveness)).ti,ab,kf. |
| 15. | (outcomes research or relative effectiveness).ti,ab,kf. |
| 16. | ((indirect or indirect treatment or mixed-treatment or bayesian) adj3 comparison*).ti,ab,kf. |
| 17. | (multi* adj3 treatment adj3 comparison*).ti,ab,kf. |
| 18. | (mixed adj3 treatment adj3 (meta-analy* or metaanaly*)).ti,ab,kf. |
| 19. | umbrella review*.ti,ab,kf. |
| 20. | (multi* adj2 paramet* adj2 evidence adj2 synthesis).ti,ab,kf. |
| 21. | (multiparamet* adj2 evidence adj2 synthesis).ti,ab,kf. |
| 22. | (multi-paramet* adj2 evidence adj2 synthesis).ti,ab,kf. |
| 23. | or/1-22 |
| 24. | exp comorbidity/ or exp multimorbidity/ or exp multiple chronic conditions/ or exp Syndemic/ |
| 25. | (Comorbid* or co-morbid* or Multimorbid* or multi-morbid* or multidisease* or multi-disease* or "multiple chronic conditions" or poly-morbidit* or polymorbidit* or poly-patholog* or polypatholog* or pluripatholog* or pluri-patholog* or multipatholog* or multi-patholog* or multicondition* or multi-condition* or syndemic or "multiple health" or "multiple chronic" or "multiple long-term" or "MLTC").mp. [mp=title, book title, abstract, original title, name of substance word, subject heading word, floating sub-heading word, keyword heading word, organism supplementary concept word, protocol supplementary concept word, rare disease supplementary concept word, unique identifier, synonyms, population supplementary concept word, anatomy supplementary concept word] |
| 26. | delphi technique/ or Consensus/ |
| 27. | (ICHOM, outcome measures or core outcome or COS or COSs or COMET or COS-STAD or measure or measurement or measuring or instrument).ti,ab. |
| 28. | 24 or 25 |
| 29. | 26 or 27 |
| 30. | 28 and 23 and 29 |
|  |  |

**Additional file 1: Table 4.** Scopus Search Strategy.

(( ABS ( ( ichom OR "delphi technique" OR "outcome measures" OR "core outcome" OR cos OR coss OR comet OR cos-stad OR measure OR measurement OR measuring OR instrument ) ) ) OR ( TITLE ( ( ichom OR "delphi technique" OR "outcome measures" OR "core outcome" OR cos OR coss OR comet OR cos-stad OR measure OR measurement OR measuring OR instrument ) ) )) AND (( ABS ( comorbid* OR co-morbid* OR multimorbid* OR multi-morbid* OR multidisease* OR multi-disease* OR "multiple chronic conditions" OR poly-morbidit* OR polymorbidit* OR poly-patholog* OR polypatholog* OR pluripatholog* OR pluri-patholog* OR multipatholog* OR multi-patholog* OR multicondition* OR multi-condition* OR syndemic OR "multiple health" OR "multiple chronic" OR "multiple long-term" OR "MLTC" ) ) OR ( TITLE ( comorbid* OR co-morbid* OR multimorbid* OR multi-morbid* OR multidisease* OR multi-disease* OR "multiple chronic conditions" OR poly-morbidit* OR polymorbidit* OR poly-patholog* OR polypatholog* OR pluripatholog* OR pluri-patholog* OR multipatholog* OR multi-patholog* OR multicondition* OR multi-condition* OR syndemic OR "multiple health" OR "multiple chronic" OR "multiple long-term" OR "MLTC" ) )) AND (TITLE-ABS-KEY ( ( systematic* W/3 ( review* OR overview* ) ) OR ( methodologic* W/3 ( review* OR overview* ) ) ) OR TITLE-ABS-KEY ( ( quantitative W/3 ( review* OR overview* OR synthes* ) ) OR ( research W/3 ( integrati* OR overview* ) ) ) OR TITLE-ABS-KEY ( ( integrative W/3 ( review* OR overview* ) ) OR ( collaborative W/3 ( review* OR overview* ) ) OR ( pool* W/3 analy* ) ) OR TITLE-ABS-KEY ( "data synthes*" OR "data extraction*" OR "data abstraction*" ) OR TITLE-ABS-KEY ( handsearch* OR "hand search*" ) OR TITLE-ABS-KEY ( "mantel haenszel" OR peto OR "der simonian" OR dersimonian OR "fixed effect*" OR "latin square*" ) OR TITLE-ABS-KEY ( "met analy*" OR metanaly* OR "technology assessment*" OR hta OR htas OR "technology overview*" OR "technology appraisal*" ) OR TITLE-ABS-KEY ( "meta regression*" OR metaregression* ) OR TITLE-ABS-KEY ( meta-analy* OR metaanaly* OR "biomedical technology assessment*" OR "bio-medical technology assessment*" ) OR TITLE-ABS-KEY ( medline OR cochrane OR pubmed OR medlars OR embase OR cinahl ) OR SRCTITLE ( cochrane OR ( health W/2 "technology assessment" ) OR "evidence report" ) OR TITLE-ABS-KEY ( comparative W/3 ( efficacy OR effectiveness ) ) OR TITLE-ABS-KEY ( "outcomes research" OR "relative effectiveness" ) OR TITLE-ABS-KEY ( ( indirect OR "indirect treatment" OR mixed-treatment OR bayesian ) W/3 comparison* ) OR TITLE-ABS-KEY ( multi* W/3 treatment W/3 comparison* ) OR TITLE-ABS-KEY ( mixed W/3 treatment W/3 ( meta-analy* OR metaanaly* ) ) OR TITLE-ABS-KEY ( "umbrella review*" ) OR TITLE-ABS-KEY ( multi* W/2 paramet* W/2 evidence W/2 synthesis ) OR TITLE-ABS-KEY ( multiparamet* W/2 evidence W/2 synthesis ) OR TITLE-ABS-KEY ( multi-paramet* W/2 evidence W/2 synthesis ))

**Additional file 1: Table 5.** Cochrane Search Strategy**.**

| #1 | MeSH descriptor: [Comorbidity] explode all trees |
| --- | --- |
| #2 | MeSH descriptor: [Multimorbidity] explode all trees |
| #3 | MeSH descriptor: [Multiple Chronic Conditions] explode all trees |
| #4 | MeSH descriptor: [Syndemic] explode all trees |
| #5 | (Comorbid* OR co-morbid* OR Multimorbid* OR multi-morbid* OR multidisease* OR multi-disease* OR "multiple chronic conditions" OR poly-morbidit* OR polymorbidit* OR poly-patholog* OR polypatholog* OR pluripatholog* OR pluri-patholog* OR multipatholog* OR multi-patholog* OR multicondition* OR multi-condition* OR syndemic OR "multiple health" OR "multiple chronic" OR "multiple long-term" OR "MLTC"):ti,ab,kw |
| #6 | #1 OR #2 OR #3 OR #4 OR #5 |
| #7 | MeSH descriptor: [Delphi Technique] explode all trees |
| #8 | MeSH descriptor: [Consensus] explode all trees |
| #9 | (ICHOM OR “outcome measures” OR “core outcome” OR COS OR COSs OR COMET OR COS-STAD OR measure OR measurement OR measuring OR instrument):ti,ab,kw |
| #10 | #7 OR #8 OR #9 |
| #11 | #6 AND #10 |

**Additional file 1: Table 6 .** CINAHL Complete Search Strategy**.**

| S10 | (S5 OR S6 OR S7) AND (S4 AND S8 AND S9) |
| --- | --- |
| S9 | S5 OR S6 OR S7 |
| S8 | S1 OR S2 OR S3 |
| S7 | ICHOM or “outcome measures” or “core outcome” or COS or COSs or COMET or COS-STAD or measure or measurement or measuring or instrument |
| S6 | (MM "Consensus") |
| S5 | (MM "Delphi Technique") |
| S4 | (MH "meta analysis" OR MH "systematic review" OR MH "Technology, Medical/EV" OR PT "systematic review" OR PT "meta analysis" OR (((TI systematic* OR AB systematic*) N3 ((TI review* OR AB review*) OR (TI overview* OR AB overview*))) OR ((TI methodologic* OR AB methodologic*) N3 ((TI review* OR AB review*) OR (TI overview* OR AB overview*)))) OR (((TI quantitative OR AB quantitative) N3 ((TI review* OR AB review*) OR (TI overview* OR AB overview*) OR (TI synthes* OR AB synthes*))) OR ((TI research OR AB research) N3 ((TI integrati* OR AB integrati*) OR (TI overview* OR AB overview*)))) OR (((TI integrative OR AB integrative) N3 ((TI review* OR AB review*) OR (TI overview* OR AB overview*))) OR ((TI collaborative OR AB collaborative) N3 ((TI review* OR AB review*) OR (TI overview* OR AB overview*))) OR ((TI pool* OR AB pool*) N3 (TI analy* OR AB analy*))) OR ((TI "data synthes*" OR AB "data synthes*") OR (TI "data extraction*" OR AB "data extraction*") OR (TI "data abstraction*" OR AB "data abstraction*")) OR ((TI handsearch* OR AB handsearch*) OR (TI "hand search*" OR AB "hand search*")) OR ((TI "mantel haenszel" OR AB "mantel haenszel") OR (TI peto OR AB peto) OR (TI "der simonian" OR AB "der simonian") OR (TI dersimonian OR AB dersimonian) OR (TI "fixed effect*" OR AB "fixed effect*") OR (TI "latin square*" OR AB "latin square*")) OR ((TI "met analy*" OR AB "met analy*") OR (TI metanaly* OR AB metanaly*) OR (TI "technology assessment*" OR AB "technology assessment*") OR (TI HTA OR AB HTA) OR (TI HTAs OR AB HTAs) OR (TI "technology overview*" OR AB "technology overview*") OR (TI "technology appraisal*" OR AB "technology appraisal*")) OR ((TI "meta regression*" OR AB "meta regression*") OR (TI metaregression* OR AB metaregression*)) OR (TI meta-analy* OR TI metaanaly* OR TI "systematic review*" OR TI "biomedical technology assessment*" OR TI "bio-medical technology assessment*" OR AB meta-analy* OR AB metaanaly* OR AB "systematic review*" OR AB "biomedical technology assessment*" OR AB "bio-medical technology assessment*" OR MW meta-analy* OR MW metaanaly* OR MW "systematic review*" OR MW "biomedical technology assessment*" OR MW "bio-medical technology assessment*") OR ((TI medline OR AB medline OR MW medline) OR (TI cochrane OR AB cochrane OR MW cochrane) OR (TI pubmed OR AB pubmed OR MW pubmed) OR (TI medlars OR AB medlars OR MW medlars) OR (TI embase OR AB embase OR MW embase) OR (TI cinahl OR AB cinahl OR MW cinahl)) OR (SO Cochrane OR SO health technology assessment OR SO evidence report) OR ((TI comparative OR AB comparative) N3 ((TI efficacy OR AB efficacy) OR (TI effectiveness OR AB effectiveness))) OR ((TI "outcomes research" OR AB "outcomes research") OR (TI "relative effectiveness" OR AB "relative effectiveness")) OR (((TI indirect OR AB indirect) OR (TI "indirect treatment" OR AB "indirect treatment") OR (TI mixed-treatment OR AB mixed-treatment) OR (TI bayesian OR AB bayesian)) N3 (TI comparison* OR AB comparison*)) OR ((TI multi* OR AB multi*) N3 (TI treatment OR AB treatment) N3 (TI comparison* OR AB comparison*)) OR ((TI mixed OR AB mixed) N3 (TI treatment OR AB treatment) N3 ((TI meta-analy* OR AB meta-analy*) OR (TI metaanaly* OR AB metaanaly*))) OR (TI "umbrella review*" OR AB "umbrella review*") OR ((TI multi* OR AB multi*) N2 (TI paramet* OR AB paramet*) N2 (TI evidence OR AB evidence) N2 (TI synthesis OR AB synthesis)) OR ((TI multiparamet* OR AB multiparamet*) N2 (TI evidence OR AB evidence) N2 (TI synthesis OR AB synthesis)) OR ((TI multi-paramet* OR AB multi-paramet*) N2 (TI evidence OR AB evidence) N2 (TI synthesis OR AB synthesis)) |
| S3 | TI ( Comorbid* or co-morbid* or Multimorbid* or multi-morbid* or multidisease* or multi-disease* or "multiple chronic conditions" or poly-morbidit* or polymorbidit* or poly-patholog* or polypatholog* or pluripatholog* or pluri-patholog* or multipatholog* or multi-patholog* or multicondition* or multi-condition* or syndemic or "multiple health" or "multiple chronic" or "multiple long-term" or "MLTC" ) OR AB ( Comorbid* or co-morbid* or Multimorbid* or multi-morbid* or multidisease* or multi-disease* or "multiple chronic conditions" or poly-morbidit* or polymorbidit* or poly-patholog* or polypatholog* or pluripatholog* or pluri-patholog* or multipatholog* or multi-patholog* or multicondition* or multi-condition* or syndemic or "multiple health" or "multiple chronic" or "multiple long-term" or "MLTC" ) |
| S2 | (MM "Syndemic") |
| S1 | (MM "Comorbidity") |

**Additional file 1: Table 7.** Core Outcome Measures in Effectiveness Trials (COMET) Search Strategy.

| Disease Name: | Multimorbidity |
| --- | --- |
| Published/ Unpublished: | Both |

**Additional file 1: Table 8.** Full-text articles excluded with reasons.

| **Study** | **Title** | **Reason for exclusion** |
| --- | --- | --- |
| Alfaro-Lara, 2014 | Pharmacological treatment conciliation methodology in patients with multiple conditions | Language other than English |
| Buck, 2013 | Measuring comorbidity in cardiovascular research: a systematic review | No core outcome development |
| Eton, 2013 | A systematic review of patient-reported measures of burden of treatment in three chronic diseases | Wrong patient population |
| Evans, 2005 | Health-related quality of life of patients with diabetes and foot ulcers | Wrong study design |
| Extermann, 2000 | Measuring comorbidity in older cancer patients | No core outcome development |
| Fortin, 2004 | Multimorbidity and quality of life in primary care: A systematic review | No core outcome development |
| Ho, 2021 | Examining variation in the measurement of multimorbidity in research: a systematic review of 566 studies | Wrong outcomes |
| Hurst, 2020 | Critical review of multimorbidity outcome measures suitable for low-income and middle-income country settings: perspectives from the Global Alliance for Chronic Diseases researchers | No core outcome development |
| Johnston, 2019 | Defining and measuring multimorbidity: a systematic review of systematic reviews | No core outcome development |
| Jones, 2021 | Methods of analysing patterns of multimorbidity using network analysis: a scoping review protocol | Wrong study design |
| Lefèvre, 2014 | What do we mean by multimorbidity? An analysis of the literature on multimorbidity measures, associated factors, and impact on health services organisation | No core outcome development |
| Makovski, 2019 | Multimorbidity and quality of life: Systematic literature review and meta-analysis | No core outcome development |
| Mangin, 2016 | Making patient values visible in healthcare: A systematic review of tools to assess patient treatment priorities and preferences in the context of multimorbidity | No core outcome development |
| Menear, 2015 | The influence of comorbid chronic physical conditions on depression recognition in primary care: A systematic review | No core outcome development |
| Nothacker, 2025 | General practitioner-based interventions to reduce hospital admissions in patients with multimorbidity living at home – A rapid review | No core outcome development |
| Orwelius, 2024 | Is comorbidity alone responsible for changes in health-related quality of life among critical care survivors? A purpose-specific review | Wrong patient population |
| Pillay, 2014 | Quality of care measures in multimorbidity | No core outcome development |
| Reeve, 2014 | Impact of comorbidity on health-related quality of life after prostate cancer treatment: Combined analysis of two prospective cohort studies | Wrong study design |
| Rookes, 2024 | Effectiveness of complex behaviour change interventions tested in randomised controlled trials for people with multiple long-term conditions: Systematic review with meta-analysis | No core outcome development |
| Schulze, 2022 | Measuring the quality of care for older adults with multimorbidity: Results of the MULTIqual Project | Wrong outcomes |
| Sin, 2006 | Mortality in COPD: Role of comorbidities | No core outcome development |
| Smith, 2018 | A Core Outcome Set for Multimorbidity Research (COSmm) | Wrong study design |
| Stewart, 2004 | Outcomes research: An overview | Wrong study design |
| Sumner, 2024 | Co-designing care for multimorbidity: a systematic review | No core outcome development |
| Tadeu, 2020 | Multimorbidity and consultation time: a systematic review | No core outcome development |
| Tsigebrhan, 2023 | Co-morbid mental health conditions in people with epilepsy and association with quality of life in low- and middle-income countries: a systematic review and meta-analysis | No core outcome development |
| Tully, 2015 | Collaborative care for comorbid depression and coronary heart disease: A systematic review and meta-analysis of randomised controlled trials | No core outcome development |
| Vidyasagaran, 2024 | Core outcome sets for trials of interventions to prevent and to treat multimorbidity in low- and middle-income countries: the COSMOS study | Duplicate/pre-print |
| Xu, 2017 | Evidence on multimorbidity from definition to intervention: An overview of systematic reviews | No core outcome development |
| Zhang, 2025 | Can integrated care interventions strengthen primary care and improve outcomes for patients with chronic diseases? A systematic review and meta-analysis | No core outcome development |

**Additional file 1: Table 9.** Summary of outcome domains across all included reviews.

| **Domain** | **Outcomes Reported** | **Reviews Reporting** |
| --- | --- | --- |
| Clinical outcomes | Mortality, maternal death, severe maternal morbidity, new comorbidity, glycaemic control (HbA1c), blood pressure, body composition, adverse drug events | Beuscart et al. (2018); Lee et al. (2023); Vidyasagaran et al. (2024); Carswell et al. (2024); Huntley et al. (2012); Stirland et al. (2020); Lee et al. (2021) |
| Healthcare utilisation / system outcomes | Hospital admissions, healthcare use and costs, treatment adherence, medication use, medication adherence, process of care measures, out-of-pocket expenditure | Beuscart et al. (2018); Vidyasagaran et al. (2024); Carswell et al. (2024); Huntley et al. (2012); Stirland et al. (2020); Lee et al. (2021); Oemrawsingh et al. (2020) |
| Patient-reported outcomes (general) | Health-related quality of life, disability, self-rated health, illness perception, burden of treatment | Lee et al. (2023); Vidyasagaran et al. (2024); Carswell et al. (2024); Stirland et al. (2020); Møller et al. (2020); Oemrawsingh et al. (2020); Lee et al. (2021) |
| Patient engagement / experience | Patient activation, empowerment, engagement, self-management, patient experience with care, participation, diabetes distress | Carswell et al. (2024); Barello et al. (2024); Møller et al. (2020) |
| Mental health outcomes | Mental health (general), depression, psychological distress, severe mental illness-related outcomes | Lee et al. (2023); Stirland et al. (2020); Carswell et al. (2024) |
| Child outcomes (maternal COS) | Neonatal/child survival, neurodevelopment, quality of life, other health outcomes in children of mothers with multimorbidity | Lee et al. (2023) |
| Indices and measurement tools | Disease counts, Charlson Comorbidity Index, Adjusted Clinical Groups, RxRisk, Self-Administered Comorbidity Questionnaire, Patient Engagement Measures (PAM, PHE-S), PROMS (PETS, MULTIPleS, MTBQ, TBQ) | Huntley et al. (2012); Stirland et al. (2020); Oemrawsingh et al. (2020); Møller et al. (2020); Barello et al. (2024); Lee et al. (2021) |

**Abbreviations:** HbA1c (glycated haemoglobin); MTBQ (multimorbidity treatment burden questionnaire); MULTIPleS (multimorbidity illness perceptions scale); PAM (patient activation measure); PETS (patient experience with treatment and self-management); PHE-S (patient health engagement scale); PROMS (patient reported outcome measures); TBQ (treatment burden questionnaire); UK (United Kingdom).

**Additional file 1: Table 10.** Domain-level results of the JBI Critical Appraisal Checklist for Systematic Reviews and Research Syntheses across included reviews.

| **First Author** | **Is the review question clearly and explicitly stated?** | **Were the inclusion criteria appropriate for the review question?** | **Was the search strategy appropriate?** | **Were the sources and resources used to search for studies adequate?** | **Were the criteria for appraising studies appropriate?** | **Was critical appraisal conducted by two or more reviewers independently?** | **Were there methods to minimize errors in data extraction?** | **Were the methods used to combine studies appropriate?** | **Was the likelihood of publication bias assessed?** | **Were recommendations for policy and/or practice supported by the reported data?** | **Were the specific directives for new research appropriate?** | **Overall appraisal: Include, Exclude, or Seek further info** |
| --- | --- | --- | --- | --- | --- | --- | --- | --- | --- | --- | --- | --- |
| **Barello et al., 2024** | Y | Y | Y | Y | Y | Y | Y | Y | N | Y | Y | I |
| Beuscart et al., 2018 | Y | Y | Y | Y | Y | Y | Y | Y | N | Y | Y | I |
| Carswell et al., 2024 | Y | Y | Y | Y | Y | Y | Y | Y | N | Y | Y | I |
| Huntley et al., 2012 | Y | Y | Y | Y | Y | Y | Y | Y | N | Y | Y | I |
| Lee et al., 2021 | Y | Y | Y | Y | Y | Y | Y | Y | N | Y | Y | I |
| Lee et al., 2023 | Y | Y | Y | Y | Y | Y | Y | Y | N | Y | Y | I |
| Møller et al., 2020 | Y | Y | Y | Y | Y | Y | Y | Y | N | Y | Y | I |
| Oemrawsingh et al., 2020 | Y | Y | Y | Y | Y | Y | Y | Y | N | Y | Y | I |
| Stirland et al., 2020 | Y | Y | Y | Y | Y | Y | Y | Y | N | Y | Y | I |
| Vidyasagaran et al., 2024 | Y | Y | Y | Y | Y | Y | Y | Y | N | Y | Y | I |

Y = Yes; N = No; I = Includ
